# Modelling the requirements and benefits of mosquito control interventions in the presence of mosquito dispersal

**DOI:** 10.1186/1475-2875-11-S1-P67

**Published:** 2012-10-15

**Authors:** Angelina M Lutambi, Nakul Chitnis, Tom Smith, Melissa Penny

**Affiliations:** 1 Swiss Tropical and Public Health Institute, Socinstrasse 57, P.O. Box CH-4002 Basel, Switzerland; 2 University of Basel, Petersplatz 1, CH-4003 Basel, Switzerland; 3 Ifakara Health Institute, P.O Box 78373, Dares Salaam, Tanzania

## Background

Vector control methods are widely used as a means to control malaria, however, the role of spatial arrangement when deploying these interventions is not well known. Understanding the effects of spatial distribution and clustering of interventions on mosquito populations can provide a guide to strategically deploying interventions to effectively maximize benefits.

## Materials and methods

A recently developed discrete-space continuous-time mathematical model of mosquito population dynamics and dispersal was extended to incorporate vector control interventions of insecticide residual spraying (IRS), larviciding and insecticide treated bednets (ITNs). Model simulations were used to determine intervention deployment strategies, for certain coverage levels, which maximize the benefits of interventions.

## Results

Assuming homogeneous distribution of water resources and humans, then clustering of IRS and larviciding interventions, when only low coverage is possible, is more beneficial than random deployment. However, with moderate coverage of these interventions, there is no added benefit with clustering compared to random deployment. For low coverage of ITNs, clustering their distribution lowers the benefits. Surprisingly, with moderate coverage of ITNs then random deployment of ITNs to humans is more beneficial than clustering.

## Conclusions

There is evidence that the effectiveness of an intervention is highly dependent on its spatial distribution. Although the results presented here are based on model assumptions, the findings are useful to consider when designing modes of deployment of interventions to offer maximal benefits.

